# Exploration of a Novel Model of Intrusive Symptoms in Posttraumatic Stress Disorder Among US Veterans

**DOI:** 10.1001/jamanetworkopen.2022.3555

**Published:** 2022-03-21

**Authors:** Or Duek, Tobias R. Spiller, Arielle Rubenstein, Robert H. Pietrzak, Ilan Harpaz-Rotem

**Affiliations:** 1 Department of Psychiatry, Yale School of Medicine, New Haven, Connecticut; 2 National Center for PTSD, West Haven VA, Connecticut

## Abstract

This cross-sectional study evaluates a posttraumatic stress disorder model that divides intrusions into internally and externally cued symptoms compared with existing models.

## Introduction

Posttraumatic stress disorder (PTSD) is a heterogeneous disorder that has been plagued by a lack of clarity regarding its core symptom dimensions. While intrusions represent the core, defining symptom cluster of PTSD, 2 potentially distinct processes have recently been proposed to comprise these symptoms: internally generated symptoms (eg, flashbacks and nightmares); and externally generated symptoms (eg, physiological/emotional reactivity following trauma reminders).^1^

However, to our knowledge, no study has evaluated whether this more refined phenotypic model of intrusive symptoms provides a better empirical fit to PTSD symptom data than current models, such as the *Diagnostic and Statistical Manual of Mental Disorders* (Fifth Edition) (*DSM-5*)^2^ 4-cluster model and the 7-factor model, which proposes hybrid anhedonia and hypervigilance clusters.^3^

To address this gap, we evaluated a PTSD model that divides intrusions into internally and externally cued symptoms. We compared the fit of this model with the *DSM-5* and 7-factor models. We then examined how these 2 intrusion clusters correlated to other PTSD symptom clusters.

## Methods

This cross-sectional study was approved by the West Haven VA institutional review board with a waiver of informed consent because we used deidentified data. This study reported following the Strengthening the Reporting of Observational Studies in Epidemiology (STROBE) reporting guideline.

Two independent samples were analyzed. The first sample included veterans who completed the PTSD Checklist for *DSM-5* (PCL-5)^4^ at admission into intensive PTSD treatment at the Department of Veterans Affairs (VA) between October 2014 and September 2016. The second sample (validation) included PCL-5 records of veterans who completed the PCL-5 on entering PTSD treatment at the VA between October 2018 and September 2019. The PCL-5 assesses the 20 *DSM-5* PTSD symptoms. Cronbach α was 0.90 for sample 1 and 0.93 for sample 2.

We used latent network modeling, a framework in which latent variables are represented by nodes, and edges between them represent partial correlations (adjusted for the influence of all other nodes).^5^ We compared 3 different structural models of PTSD symptoms: (1) *DSM-5*, (2) 7-factor model, and (3) 8-factor (internally and externally cued intrusions) (eTable in the Supplement). Model fit was independently tested and compared in samples 1 and 2 (validation). All analyses were conducted in R statistical software with the Psychonetrics package version 0.9 (R Project for Statistical Computing). *P* values were 2-sided, and significance was set a α = .10. Data were analyzed from August to October 2021.

## Results

Sample 1 included 7367 veterans (median [IQR] age, 44.1 [33.3-55.1] years, 6255 [85%] men). Sample 2 included 52 609 PCL-5 records (median [IQR] age, 44.7 [35.3-56.8] years; 45 193 [86%] men). The mean (SD) PCL-5 score was 59.1 (11.9) in sample 1 and 57.8 (12.5) in sample 2. The proposed 8-factor model (separating the intrusion cluster into internal and external) had significantly superior fit than the *DSM-5* and 7-factor models (χ^2^ difference: sample one, 3811; sample two, 822; *P* < .001) (Table). Fit testing in the validation sample yielded comparable results, with the 8-factor model evidencing the best fit (Table). Network analysis revealed positive partial correlations between externally cued intrusions and avoidance symptoms and between internally cued intrusions and dysphoric arousal symptoms (Figure).

**Table.** Intrusive Symptom Mappings and Fit Indices of the *DSM-5*, 7-Factor Hybrid, and 8-Factor Structural Models of PTSD Symptoms

Measure	*DSM-5* Model	7-Factor model	8-Factor model
**Sample 1 (n = 7367)**			
TLI	0.87	0.94	0.96
CFI	0.89	0.95	0.97
RMSEA (90% CI)	0.074 (0.072-0.075)	0.050 (0.048-0.051)	0.042 (0.040-0.043)
χ^2^	6809	2997	2175
BIC	360 695	356 964	356 150
AIC	360 253	356 460	355 639
*df*	166	157	156
**Sample 2 (n = 52 609)**			
TLI	0.90	0.95	0.96
CFI	0.91	0.96	0.97
RMSEA (90% CI)	0.070 (0.069-0.070)	0.050 (0.050-0.051)	0.042 (0.042-0.043)
χ^2^	42 215	20 108	13 841
BIC	245 7264	243 5298	242 9085
AIC	2 456 679	2 434 598	242 8340
*df*	164	151	146

Abbreviations: AIC, Akaike information criterion; CFI, comparative fit index; *DSM-5*, *Diagnostic and Statistical Manual of Mental Disorders (Fifth Edition)*; BIC, bayesian information criterion; RMSEA, root mean square error of approximation; TLI, Tucker-Lewis index.

**Figure.**
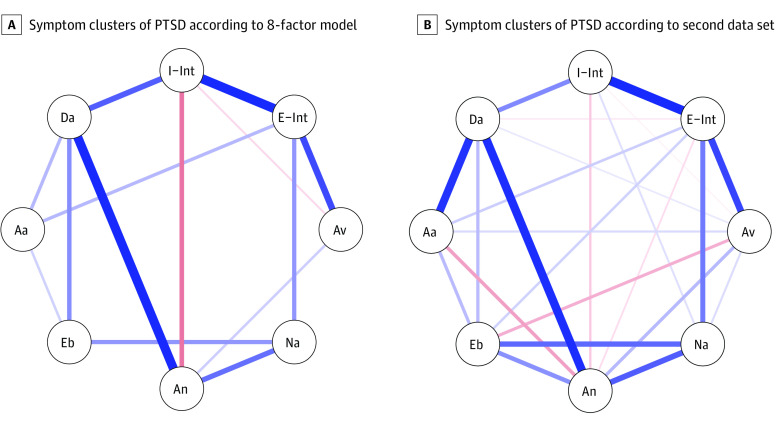
Partial Correlations Between the Different Symptom Clusters of PTSD Comprised of Internally (I-) and Externally Cued (E-) Intrusive Symptoms (Int) Blue lines represent positive edges and red lines represent negative edges. The width and hue of the lines indicate relative edge strength. Aa indicates anxious arousal; An, anhedonia; Av, avoidance; Da, dysphoric arousal; Eb, externalizing behavior; and Na, negative affect.

## Discussion

This cross-sectional study provides the first empirical support, to our knowledge, for a recently proposed conceptual distinction between internally and externally generated intrusive symptoms of PTSD.^1^ Latent network analyses revealed a superior fit of a theory-driven model with 2 separate intrusion clusters, as well as distinct connections between these 2 clusters, and other PTSD symptom clusters. Specifically, externally cued intrusions were associated with avoidance behaviors, while internally generated intrusions were associated with dysphoric arousal.

This distinction between intrusive symptoms may have clinical implications. For example, externally cued intrusions may have fear-based origins that respond better to exposure therapy, while internally generated intrusions may be linked to more cognitive-ruminative processes and thus, will need a different therapeutic approach.

While the cross-sectional and veteran-specific samples limit the generalizability of this study, results suggest that separating intrusive symptoms may provide a more nuanced phenotypic representation of the phenotypic expression of PTSD. Further research is needed to examine neurobiological underpinnings of these symptom clusters, and their utility in informing the prognosis of PTSD and precision medicine-based interventions for this disorder.

## Notes

Supplement. **eTable. **Mappings of PTSD Checklist for *DSM-5* Items for *DSM-5*, 7-Factor, and 8-Factor Structural Models
