# Supplementary material for: Exploration of a Novel Model of Intrusive Symptoms in Posttraumatic Stress Disorder Among US Veterans
Source: JAMA Netw Open. 2022 Mar 21;5(3):e223555. doi: 10.1001/jamanetworkopen.2022.3555 (PMC8938717; doi:10.1001/jamanetworkopen.2022.3555)
Supplement: Supplement. — eTable. Mappings of PTSD Checklist for DSM-5 Items for DSM-5, 7-Factor, and 8-Factor Structural Models [file jamanetwopen-e223555-s001.pdf]

Supplemental Online Content

Duek O, Spiller TR, Rubenstein A, Pietrzak RH, Harpaz-Rotem I. Exploration of a novel model of intrusive symptoms in posttraumatic stress disorder among US veterans. *JAMA Netw Open*. 2022;5(3):e223555. doi:10.1001/jamanetworkopen.2022.3555

eTable. Mappings of PTSD Checklist for *DSM-5* Items for *DSM-5*, 7-Factor, and 8-Factor Structural Models

This supplemental material has been provided by the authors to give readers additional information about their work.

© 2022 Duek O et al. *JAMA Network Open.*

eTable. Mappings of PTSD Checklist for *DSM-5* Items for *DSM-5*, 7-Factor, and 8-Factor Structural Models

DSM-5	7-factor	8-factor	Item No.	
Int	*Int*	I-Int	1	Repeated, disturbing, and unwanted memories of the stressful experience
Int	*Int*	I-Int	2	Repeated, disturbing dreams of the stressful experience
Int	*Int*	I-Int	3	Suddenly feeling or acting as if the stressful experience were actually happening again (as if you were actually back there reliving it)
Int	*Int*	E-Int	4	Feeling very upset when something reminded you of the stressful experience
Int	*Int*	E-Int	5	Having strong physical reactions when something reminded you of the stressful experience (for example, heart pounding, trouble breathing, sweating)
AV	*AV*	*AV*	6	Avoiding memories, thoughts, or feelings related to the stressful experience
AV	*AV*	*AV*	7	Avoiding external reminders of the stressful experience (for example, people, places, conversations, activities, objects, or situations)
NACM	*NA*	*NA*	8	Trouble remembering important parts of the stressful experience
NACM	*NA*	*NA*	9	Having strong negative beliefs about yourself, other people, or the world (for example, having thoughts such as: I am bad, there is something seriously wrong with me, no one can be trusted, the world is completely dangerous)
NACM	*NA*	*NA*	10	Blaming yourself or someone else for the stressful experience or what happened after it
NACM	*NA*	*NA*	11	Having strong negative feelings such as fear, horror, anger, guilt, or shame
NACM	*AN*	*AN*	12	Loss of interest in activities that you used to enjoy
NACM	*AN*	*AN*	13	Feeling distant or cut off from other people
NACM	*AN*	*AN*	14	Trouble experiencing positive feelings (for example, being unable to feel happiness or have loving feelings for people close to you)
Hyper	*EB*	*EB*	15	Irritable behavior, angry outbursts, or acting aggressively
Hyper	*EB*	*EB*	16	Taking too many risks or doing things that could cause you harm
Hyper	*AA*	*AA*	17	Being “superalert” or watchful or on guard
Hyper	*AA*	*AA*	18	Feeling jumpy or easily startled
Hyper	*DA*	*DA*	19	Having difficulty concentrating
Hyper	*DA*	*DA*	20	Trouble falling or staying asleep

Abbreviations: *Int*: Intrusion symptoms. *AV:* Avoidance. NA: Negative affect. AN: Anhedonia. EB: Externalizing behaviors. *AA*: Anxious arousal. *DA*: Dysphoric arousal. *NACM*: Negative alterations in cognitions and mood. *Hyper*: Alterations in arousal and reactivity. *I-Int*: Internally-generated intrusion symptoms. *E-Int*: Externally-generated intrusion symptoms.

© 2022 Duek O et al. *JAMA Network Open.*
